# Green and Short Preparation of CeO_2_ Nanoparticles with Large Specific Surface Area by Spray Pyrolysis

**DOI:** 10.3390/ma14174963

**Published:** 2021-08-31

**Authors:** Yongfu Wu, Hong Li, Xue Bian, Wenyuan Wu, Zhenfeng Wang, Yubao Liu

**Affiliations:** 1 School of Metallurgy, Northeastern University, Shenyang 110819, China; wyf07@imust.edu.cn (Y.W.); wuwy@smm.neu.edu.cn (W.W.); 2 School of Energy and Environment, Inner Mongolia University of Science and Technology, Baotou 014010, China; lhong25@163.com; 3 School of Energy and Civil Engineering, Shandong HuaYu University of Technology, Dezhou 253034, China; 4 State Key Laboratory of Baiyunobo Rare Earth Resource Researches and Comprehensive Utilization, Baotou Research Institute of Rare Earths, Baotou 014030, China; liuyubao1985@126.com

**Keywords:** spray pyrolysis, CeO_2_, specific surface area, spray pyrolysis temperature, citric acid

## Abstract

Green and short preparation of CeO_2_ nanoparticles with large specific surface area from rare earth extraction (CeCl_3_) was successfully achieved by spray pyrolysis (SP). In this method, a precursor solution is first prepared by mixing CeCl_3_, C_6_H_8_O, and H_2_O in the requisite quantities. Subsequently, the precursor consisting of a mixture of CeO_2_ and C was obtained by SP method by using the precursor solution. Finally, the calcination at 500 °C~800 °C in air for two hours to transform the precursor to CeO_2_ nanoparticles. Thermodynamic analysis and experimental studies were performed to determine the optimal SP temperature and citric acid amount. The results indicated that the maximum specific surface area (59.72 m^2^/g) of CeO_2_ nanoparticles were obtained when the SP temperature was 650 °C and the molar ratio of citric acid to CeCl_3_ was 1.5.

## 1. Introduction

Cerium oxide (CeO_2_) is a widely used rare earth oxide material. In China, limited application of industrially-produced CeO_2_ leads to its high output and low usage. Nanoscale CeO_2_ with a large specific surface area retains the unique crystal structure of CeO_2_ and exhibits a series of other functional properties. It can be used to treat automobile exhaust [1,2], purify organic wastewater [3,4], as a raw material in solid-state fuel batteries [5,6], and anti-tumor biomedicine [7].

The common preparation methods of nanoscale CeO_2_ include precipitation, hydrothermal synthesis, and sol-gel methods, which increase the specific surface area by controlling the nanoparticle growth. Shih C J. et al. [8] CeO_2_ crystals were synthesized by using a co-precipitation method in an aqueous solution at a relatively low temperature and it was concluded that the crystal grain size increased with increasing temperature. Muduli S K. et al. [9] Ethylene glycol and isopropanol were mixed as solvents with cerium ammonium nitrate solution to synthesize CeO_2_ with a specific surface area of 93 m^2^/g via solvothermal method. Zhou et al. [10] Cerium nitrate was used as the cerium source and polyethylene pyrrolidone (PVP) was used as a surfactant to hydrothermally synthesize spherical CeO_2_ nanoparticles. The specific surface area was controlled by changing the cerium nitrate concentration and the molar ratio of PVP to cerium nitrate. Mudler et al. [11] Cerium acetate was dissolved in glacial acetic acid and a mixture of isooctane and 2-butanol was added to obtain highly-crystalline CeO_2_. Various specific surface areas were achieved by controlling the flow rate of oxygen and raw material in a flame spray pyrolysis method. Katalenich et al. [12] Taking organic cerium ammonium nitrate, hexamethylenetetramine (HMTA), urea, and ammonium hydroxide as the raw materials, a sol-gel method was used to prepare spherical CeO_2_ particles with an average size of 100 μm. The specific surface area of particles was also increased by changing their porosity, as reported in Refs. [13,14,15]. However, the above methods require cumbersome steps and long preparation time, which greatly limit the preparation efficiency and increase the preparation cost.

Spray pyrolysis of rare earth extract (ReCl_3_) is an effective method to realize green and short process preparation of rare earth materials. Spray pyrolysis, which involves evaporating a CeCl_3_ solution in a furnace, solute precipitation, drying, metal salt decomposition, sintering, etc., can achieve continuous production. [16,17] The steps of preparation are simple, the time is short, and the chemical uniformity is controllable. In addition, this method ensures the independent growth of particles among atomized droplets and allows a narrow particle size distribution. In comparison with previous research which has indicated that spray pyrolysis cannot sufficiently control the morphology and structure of CeO_2_, this method can be used to quickly and efficiently prepare CeO_2_ [18,19]. The size of the prepared CeO_2_ particles can reach the micro-nano scale, but the occurrence of serious agglomeration reduces the specific surface area. The water and CO_2_ produced by the decomposition of citric acid do not undergo side reactions during the pyrolysis of cerium chloride. Hence, an optimal concentration of citric acid is required to control the surface tension of the solution. The atomized droplets are reduced, thereby the particle size of the product is effectively controlled [20].

In this paper, a spray pyrolysis method was used to directly prepare CeO_2_ nanoparticles with a large surface area from CeCl_3_. The optimal pyrolysis temperature and the ratio of citric acid were determined. The results may provide technical support for the preparation of CeO_2_ nanoparticles with large surface areas.

## 2. Experimental

### 2.1. Experimental System

**Figure 1:**
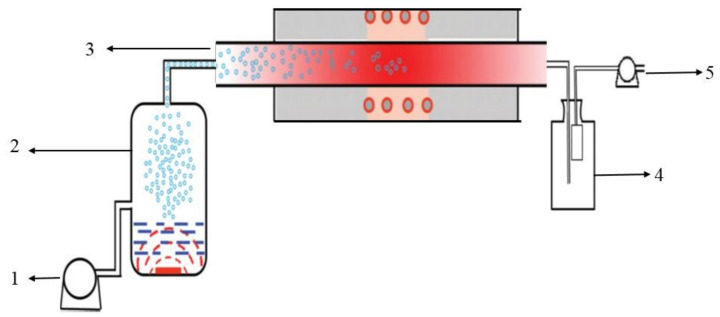
Schematic diagram of spray pyrolysis equipment. (**1**) Air compressor; (**2**) Liquid storage bottle; (**3**) Reaction furnace; (**4**) Collection bottle; (**5**) Recovery device.

A schematic illustration of the spray pyrolysis equipment used in the experiment is shown in Figure 1. Air pump 1 provides high-speed air to carry mixed droplets of cerium chloride and citric acid generated in atomization chamber 2; the mixed droplets pass through heating furnace 3 for pyrolysis; particle collection device 4 collects pyrolysis products CeO_2_ and carbon; the tail gas is collected by the recovery device 5.

### 2.2. Instrument and Materials

Cerium chloride (CeCl_3_∙7H_2_O) with a purity of 99.99% provided by Jining Tianyi New Material Co., Ltd. in Jining, Shandong, was used as raw material. Citric acid (C_6_H_8_O_7_∙H_2_O) with purity greater than 99.5% provided by Tianjin Fengchuan Chemical Reagent Technology Co., Ltd. in Tianjin, China was used as the additive. Deionized water was used as the solvent of the solution to prepare the precursor solution.

The equipment used in the experiments includes X-ray powder diffractometer (XRD, Japan, Miniflex600 desktop X-ray diffractometer, Cu Kα rays); Dual-ion field emission scanning electron microscope (SEM, FIB/FE-SEM GAIA 3 XMN); High-resolution transmission electron microscope (TEM, Japan, JEM-2100); and Specific surface area and pore size analyzer (BET, Beishide Instrument Technology (Beijing) Co., Ltd., 3H-2000PS1 static volume method). Eleven replicates of experimental runs for the specific surface area were performed in this study.

### 2.3. Experimental Procedures

CeCl_3_∙7H_2_O was first dissolved in DI water to prepare a 30% CeCl_3_ solution, and then a certain amount of citric acid (C_6_H_8_O_7_) was added in the CeCl_3_ solution with different molar ratios of citric acid to CeCl_3_ (A/C) of 0, 0.5, 1, 1.5, 2, and 2.5 to form the precursor solution. The spray pyrolysis furnace was heated until its temperature reached the rated value, and the precursor solution in the liquid storage bottle was atomized into small droplets through a nozzle and sprayed into the high-temperature zone of the furnace under the action of 0.3 MPa carrier gas. The furnace temperature was set as 500, 650, 750, and 850 °C to prepare the pyrolysis products of CeO_2_ and carbon. The products were collected when they reached the cyclone separator collection location under the downward force of a fan. The collected samples were calcined in a muffle furnace at 700 ℃ in air for 2 h to remove the residual carbon and to obtain the CeO_2_ products in accordance with Equation (1).

C + O_2_→CO_2_ (1)

These obtained samples were characterized by XRD, SEM, TEM, and BET. The test conditions of the experiment are shown in Table 1. Cross-experiments were carried out for two variables.

## 3. Results and Discussion

### 3.1. Thermodynamic Analysis

According to the reaction Equation (2), the Gibb’s free energy of CeCl_3_ to prepare CeO_2_ was calculated to assess the feasibility of the reaction.

4CeCl_3_(l) + O_2_(g) + 6H_2_O(l) = 4CeO_2_ + 12HCl(g) (2)

**Figure 2:**
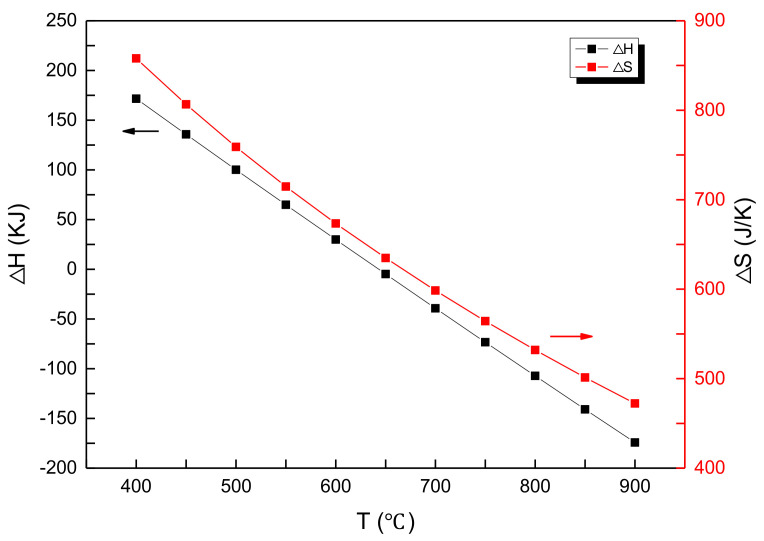
The dependence of ΔH and ΔS of Equation (2) with reaction temperature.

**Figure 3:**
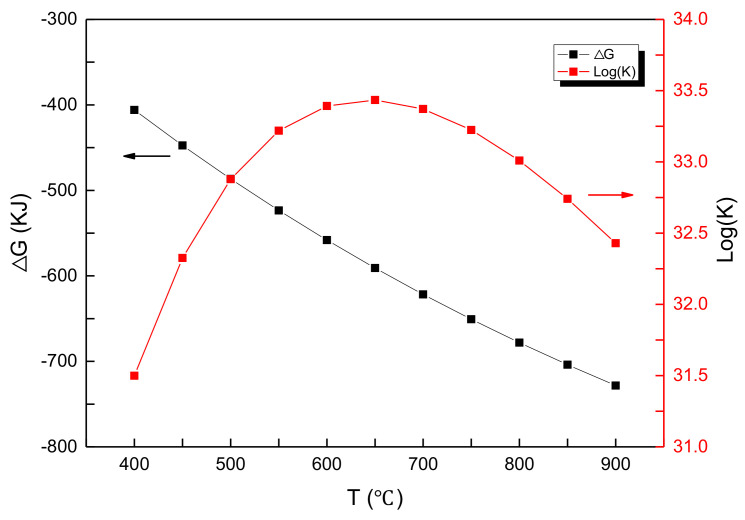
The dependence of ΔG and log(K) of Equation (2) with reaction temperature.

Thermodynamic analysis of the reaction Equation (2) was carried out by the HSC Chemistry 6 thermodynamic simulation software. The reaction enthalpy (ΔH), reaction entropy(ΔS), Gibb’s free energy(ΔG), and equilibrium constant (log(K)) of the reaction (2) are shown in Figure 2 and Figure 3, respectively. The Gibb’s free energy decreases with the temperature, indicating that the reaction is likely to occur at higher temperatures (Figure 2). The chemical equilibrium constant increases and then decreases, showing a maximum at 650 °C, indicating that the reaction proceeds completely at 650 °C (Figure 3).

### 3.2. Characterization by XRD and SEM

**Figure 4:**
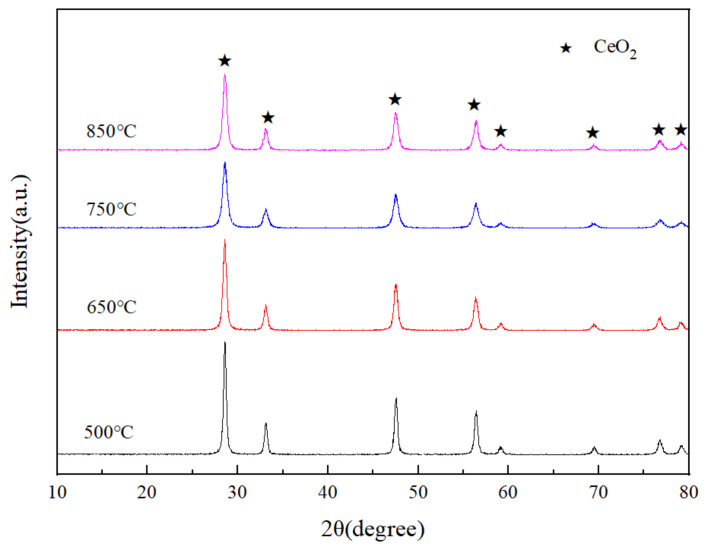
XRD patterns of the CeO_2_ products prepared at various spray pyrolysis temperatures.

Figure 4 shows the XRD patterns of the CeO_2_ products prepared at various spray pyrolysis temperatures. Apparently, four CeO_2_ products exhibit the same peaks assigned to CeO_2_, as shown in Figure 4. However, the intensities of CeO_2_ peaks of four CeO_2_ products show a light difference. The difference may be ascribed to the multiple effects of heat generated from the combustion of the residual carbon during calcination and the spray pyrolysis temperature. However, the comprehension about the difference for the diffraction intensity of these CeO_2_ products is still under investigation and will be published later.

**Figure 5:**
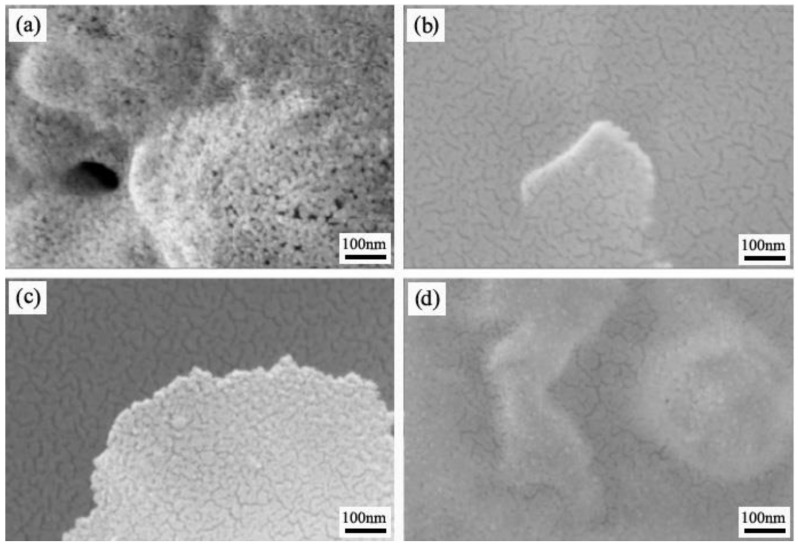
SEM images of the CeO_2_ products prepared at various spray pyrolysis temperatures (**a**) 500 °C; (**b**) 650 °C; (**c**) 750 °C; (**d**) 850 °C.

The CeO_2_ products were characterized by SEM, as shown in Figure 5. The CeO_2_ products prepared at various different spray pyrolysis temperatures were agglomerated CeO_2_ nanoparticles. The particle size of CeO_2_ decreases and then increases with the spray pyrolysis temperature, showing the minimum (about 15 nm) at 650 °C, which indicates that the spray pyrolysis temperature affects the size of CeO_2_ nanoparticles.

### 3.3. Analysis of Specific Surface Area and Particle Size

**Figure 6:**
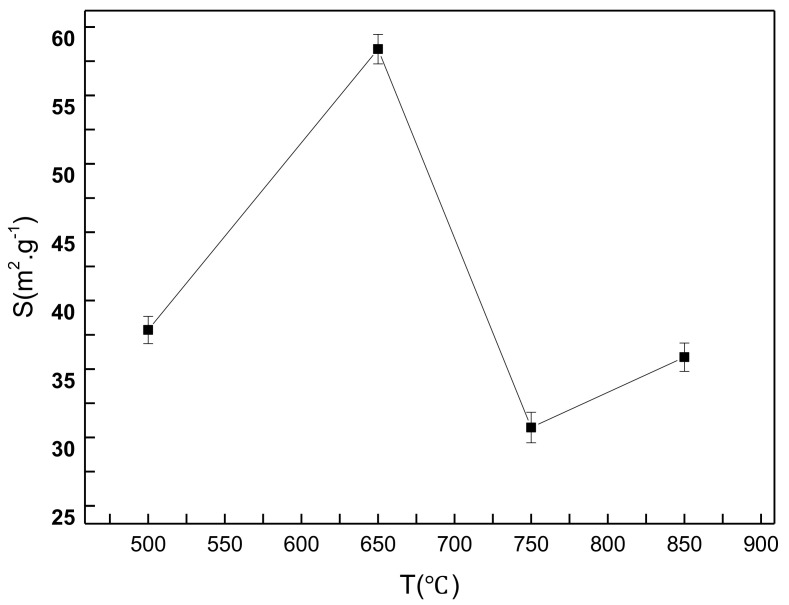
The specific surface area of the CeO_2_ products prepared at various spray pyrolysis temperatures.

Figure 6 presents the specific surface area as a function of spray pyrolysis temperature for the CeO_2_ products. Here, error bars in Figure 6 show standard deviations for eleven replicates of experimental runs. It is obvious in Figure 6 that the specific surface area of CeO_2_ increases and then decreases with increasing the spray pyrolysis temperature, and reaches a maximum of 59.72 m^2^/g at 650 °C. Combined with the XRD results, it can be concluded that CeO_2_ formed at low temperatures, but with a small specific surface area. At higher temperatures, the reaction was fast, the precursor solution quickly evaporated, and the pyrolysis products of CeO_2_ and residue carbon formed. Subsequently, the pyrolysis product was calcined in the muffle furnace to remove the residue carbon and to obtain the CeO_2_ products. A large amount of heat was generated in the combustion of the residue carbon during calcination, decreasing the specific surface area. Therefore, the optimal spray pyrolysis temperature for preparing CeO_2_ is found to be 650 °C.

### 3.4. Effects of Citric Acid

**Figure 7:**
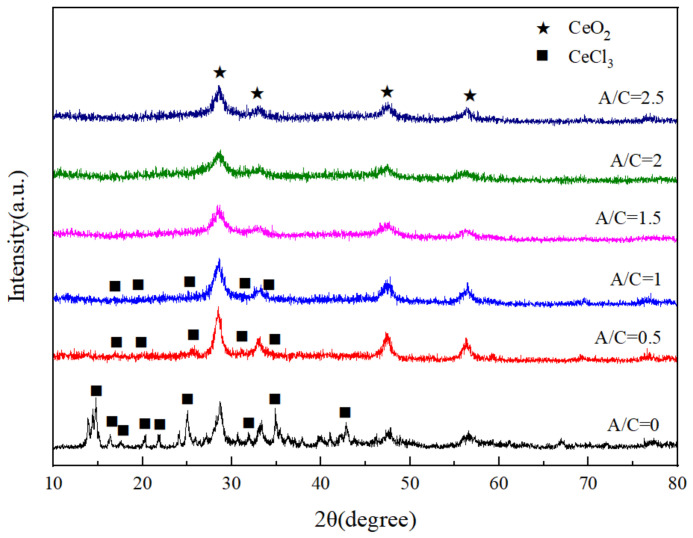
XRD patterns of the pyrolysis products prepared with various (A/C).

X-ray diffraction (XRD) patterns of six pyrolysis products, prepared with various (A/C), are presented in Figure 7. Apparently, six pyrolysis products exhibit the main peaks assigned to CeO_2_. However, the XRD peaks of six pyrolysis products show a light difference. It is obvious from Figure 7 that the pyrolysis products; prepared with (A/C = 0, 0.5, and 1), exhibit extra CeCl_3_ peak apart from peaks of CeO_2_. Therefore, it can be assumed that the pyrolysis products prepared with (A/C = 1.5, 2, and 2.5) completed the pyrolysis reaction, while this reaction for the pyrolysis products prepared with (A/C = 0, 0.5, and 1) is incomplete under the similar conditions. It indicates that the effect of citric acid amount on the percent conversion from CeCl_3_ to CeO_2_. Furthermore, XRD intensity of CeO_2_ peak for the the pyrolysis products prepared with (A/C = 1.5, 2, and 2.5) is higher than that of the pyrolysis products prepared with (A/C = 0, 0.5, and 1).

**Figure 8:**
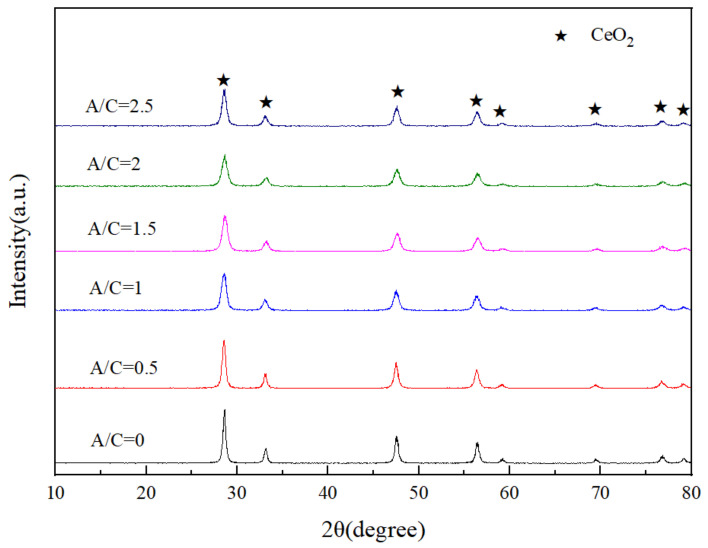
XRD patterns of the CeO_2_ products prepared with various (A/C).

Figure 8 displays the diffraction patterns of the CeO_2_ products prepared with various (A/C). The diffraction intensity of the CeO_2_ products is greatly improved compared with the corresponding pyrolysis products (Figure 7). Each diffraction peak of CeO_2_ is relatively clear, indicating good-crystallization of the samples.

**Figure 9:**
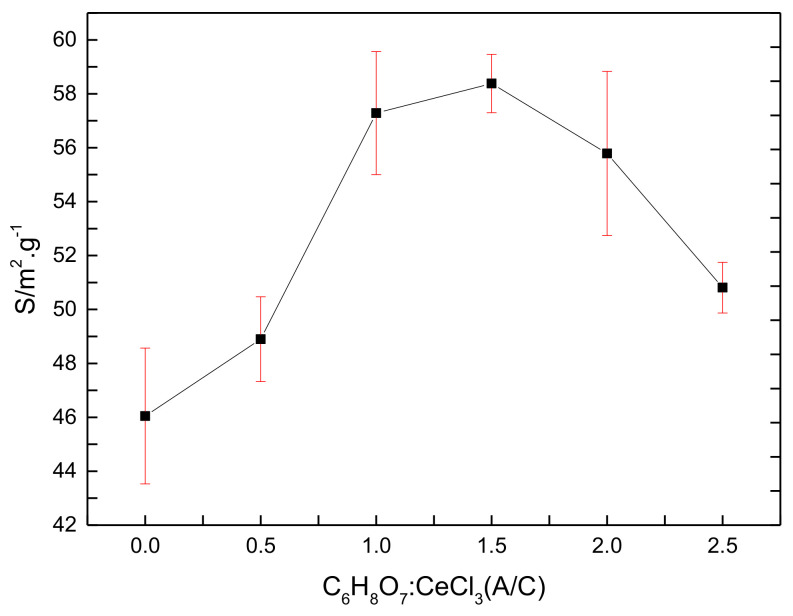
The specific surface area of the CeO_2_ products prepared with various (A/C).

Figure 9 shows the specific surface area of the CeO_2_ products prepared with various (A/C). Here, error bars in Figure 9 show standard deviations for eleven replicates of experimental runs. It is obvious from Figure 9 that the specific surface area increases and then decreases as the (A/C) increases. The specific surface area reaches a maximum of 59.72 m^2^/g when the (A/C) is 1.5. It is because the decomposition of citric acid in the heating furnace produced a large amount of gas, and the increased pressure caused the spherical CeO_2_ to crack, thereby its specific surface area increased. When the (A/C) was increased, the residual amorphous carbon increased, and the combustion of the residual carbon released a large amount of heat, leading to a decrease in the specific surface area. It indicates that citric acid increases the specific surface area of the CeO_2_ products, but a high citric acid content (such as A/C = 2 and 2.5) inhibits an increase in the specific surface area.

**Figure 10:**
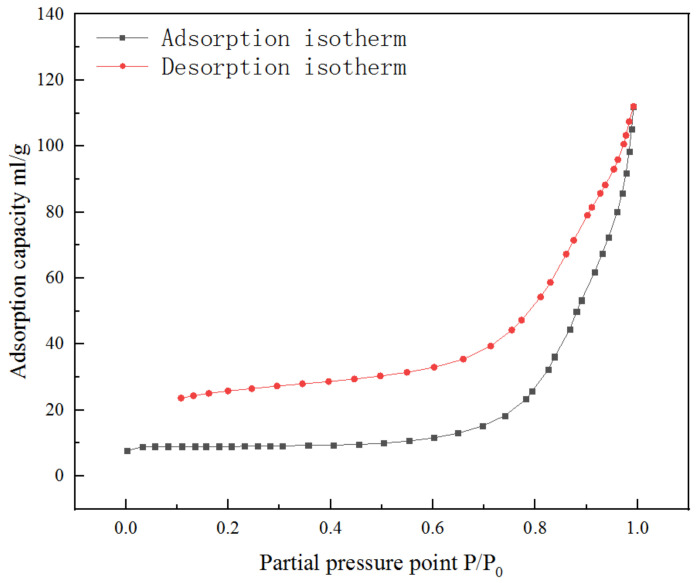
Adsorption-desorption isotherm of the CeO_2_ product prepared with A/C = 1.5.

**Figure 11:**
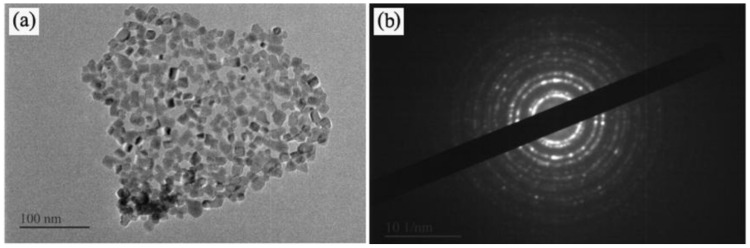
TEM images of the CeO_2_ product prepared with (A/C = 1.5). (**a**) Typical TEM images; (**b**) SAED diffraction ring.

Figure 10 shows the nitrogen adsorption curve of the CeO_2_ product prepared with A/C = 1.5. From the adsorption-desorption isothermal line, it can be known that it is a type IV isotherm, which represents non-porous materials. The adsorption volume in the low pressure area increases gently with the increase in P/P_0_, and the separation of capillary condensation appears in the high-pressure zone, indicating that the sample is solid nanoparticles, which is consistent with the results of transmission electron microscopy (TEM) (Figure 11).

Figure 11 shows the TEM image of the CeO_2_ product with (A/C = 1.5). The CeO_2_ product exhibits well-distributed spherical particles ranging from 15 to 20 nm (Figure 11a). This confirms that the spray pyrolysis method can be used to produce CeO_2_ nanoparticles. Multiple concentric diffraction halos are seen in the SAED diffraction ring in Figure 11b, indicating that the CeO_2_ nanoparticles are polycrystalline.

## 4. Conclusions

CeO_2_ nanoparticles were successfully synthesized by combining spray pyrolysis and calcination routes. This green and short process involves the preparation of a pyrolysis product by spray pyrolysis method, followed by the calcination reaction. The largest specific surface area of CeO_2_ was obtained at a pyrolysis temperature of 650 °C. The specific surface area first increased and then decreased as the A/C gradually increased. The specific surface area reached a maximum of 59.72 m^2^/g when the A/C was 1.5. The results provide a green and feasible method for the direct preparation of cerium oxide nanomaterials from rare earth cerium chloride solution.

## Figures and Tables

**Table 1:** Test conditions.

Spray Pyrolysis Temperature (°C)		C_6_H_8_O_7_: CeCl_3_ (A/C)				
	0	0.5	1	1.5	2	2.5
500				√		
650	√	√	√	√	√	√
750				√		
850				√		

## Data Availability

The data presented in this study are available on request from the corresponding author.
